# Unraveling the Pathogenesis of Calcinosis in Systemic Sclerosis: A Molecular and Clinical Insight

**DOI:** 10.3390/ijms252011257

**Published:** 2024-10-19

**Authors:** Aslihan Avanoglu Guler, Giacomo De Luca, Lorenzo Dagna, Marco Matucci-Cerinic, Corrado Campochiaro

**Affiliations:** 1 Unit of Rheumatology, Ankara Etlik City Hospital, 06010 Ankara, Turkey; aslihanavanoglu@gmail.com; 2 Unit of Immunology, Rheumatology, Allergy and Rare Diseases, IRCCS San Raffaele Hospital, Vita-Salute San Raffaele University, 20132 Milan, Italy; deluca.giacomo@hsr.it (G.D.L.); dagna.lorenzo@unisr.it (L.D.); matuccicerinic.marco@hsr.it (M.M.-C.)

**Keywords:** calcinosis, clinical features, complications, dynamin-related protein-1, extracellular matrix, mitochondrial fission, osteogenic differentiation, systemic sclerosis

## Abstract

Dystrophic calcinosis, which is the accumulation of insoluble calcified crystalline materials within tissues with normal circulating calcium and phosphorus levels, is a frequent finding in systemic sclerosis (SSc) and represents a major burden for patients. In SSc, calcinosis poses significant challenges in management due to the associated risk of severe complications such as infection, ulceration, pain, reduction in functional capacity and quality of life, and lack of standardized treatment choices. The exact pathogenesis of calcinosis is still unknown. There are multifaceted factors contributing to calcinosis development, including osteogenic differentiation of cells, imbalance between promoter and inhibitors of mineralization, local disturbance in calcium and phosphate levels, and extracellular matrix as a template for mineralization. Several pathophysiological changes observed in SSc such as ischemia, exacerbated production of excessive reactive oxygen species, inflammation, production of inflammatory cytokines, acroosteolysis, and increased extracellular matrix production may promote the development of calcinosis in SSc. Furthermore, mitochondrial dynamics, particularly fission function through the activity of dynamin-related protein-1, may have an effect on the dystrophic calcinosis process. In-depth investigations of cellular mechanisms and microenvironmental influences can offer valuable insights into the complex pathogenesis of calcinosis in SSc, providing potential targeting pathways for calcinosis treatment.

## 1. Introduction

Systemic sclerosis (SSc) is a chronic autoimmune connective tissue disease characterized by complex pathogenesis, including inflammation, vasculopathy, and fibrosis [1]. Given the link between major organ involvements of SSc and the increased SSc-related mortality rates, certain clinical manifestations such as calcinosis might be overlooked [2]. However, dystrophic calcinosis is a prevalent finding in SSc patients, with higher frequency ranging from 18 to 49% [3]. Additionally, calcinosis leads to severe complications such as infection and fistulation in patients with digital ulcers, intractable pain, and functional disability, all of which might result in a reduction in quality of life and severe morbidities in SSc patients [4,5,6]. The management and treatment of calcinosis in SSc are not standardized and are usually based on expert opinions and reports derived from observational or non-randomized controlled studies. Although the exact pathophysiology of calcinosis in SSc is still unknown, hypoxia/ischemia, mechanical stress, and microtraumas are considered possible contributors to the development of calcinosis in SSc [7]. In this review, we aim to assess the association between pivotal factors contributing to the development of calcinosis and the pathophysiological changes observed in SSc patients, thus providing potential causative factors underlying calcinosis in SSc.

## 2. The Process of Crystal Formation in Systemic Sclerosis

The process of crystal formation begins with the nucleation phase in which molecules and ions accumulate and this stage is followed by crystal growth [8]. The development and growth of pathologic crystals in tissue is not a simple process. It depends on several factors, including the level of solute supersaturation, considered a main initiator of crystallization; a reduction in mineralization inhibitors; an increase in mineralization promoters; and the presence of extracellular matrix and cells. Dystrophic calcification or calcinosis observed in SSc patients denotes the accumulation of insoluble calcified crystalline materials within tissues with normal circulating calcium and phosphorus levels. Hydroxyapatite (Ca_10_(PO_4_)_6_(OH)_2_), a mineralized form of calcium phosphate crystal, is the main crystal component of dystrophic calcinosis in SSc and aggregates in the form of micro- and macro-deposits [9,10,11]. The analyses of calcinosis components from 10 SSc patients have shown that the only detected crystalline with X-ray diffraction is hydroxyapatite, constituting fewer than 50% of most samples [12]. Furthermore, the chemical characterization of calcified deposits with vibrational spectroscopy demonstrated that dystrophic calcinosis cutis is composed of type B hydroxyapatite carbonate, the predominant biologic apatite in bones [13,14]. We hypothesize that the development of calcinosis in SSc may be explained by three main steps: the osteogenic differentiation of cells induced by several pathways and conditions, a local increase in phosphate (PO_4_^3−^) and calcium (Ca^+2^) levels provided by apoptotic cells, and osteoclast activation and collagen fibers/extracellular matrix as a template.

### 2.1. Osteogenic Differentiation of Cells

Mesenchymal stem/stromal cells (MSCs) have a crucial impact on tissue homeostasis through their immunoregulatory effects; tissue regeneration/repairing; and capacity of differentiation into various cells such as adipocytes, chondrocytes, stromal cells, and osteoblasts [15,16]. The potential differentiation of MSCs is influenced by many factors, including microenvironment conditions, growth factors, and cytokines. The evaluation of MSCs derived from adipose tissue in SSc has highlighted that SSc-related MSCs exhibit similar phenotype and differentiation behaviors, including osteophenotype, compared to those from healthy controls [17]. However, sera from SSc patients have revealed a remarkable effect on the osteogenic differentiation of MSCs. MSCs are significantly more prone to osteogenic capacity with SSc-derived sera including higher oxidative stress in contrast to healthy sera treated with oxidative stress [18]. Another in vitro study has shown that adding interstitial fluid and macrophages from SSc patients leads to the production of calcium deposits in MSCs derived from adipose tissue. Notably, this process can be suppressed by the repolarization of macrophages and the inhibition of transforming growth factor-beta (TGF-β) [19]. Pro-inflammatory cytokines, including interleukin-1 (IL-1), IL-6, and tumor necrosis factor-alpha (TNF-α), exert an additive influence on the differentiation of MSCs derived from adipose tissue into osteoblastic activity and calcification through the induction of Runx2, which is the key transcription factor for osteoblastogenesis. Moreover, it has been demonstrated that IL-6 has a major impact on the calcinosis process [20].

Dermal fibroblasts and fibroblasts from peripheral blood are capable of differentiation into osteoblast-like phenotype cells and produce calcium deposits when exposed to an osteogenic medium containing dexamethasone, ascorbic acid, and b-glycerophosphate [21,22]. Myofibroblasts, the principal cells involved in the fibrosis mechanism of SSc, primarily originate from fibroblast activation, but they can also be derived from pericytes, endothelial cells, and adipose cells [23]. Myofibroblasts, characterized by the presence of alpha-smooth muscle actin (α-SMA), exhibit osteogenic behavior when derived from fibroblast induced with elastin-derived peptides (EDPs) and TGF-β1, both of which are increased in SSc patients [24,25].

Endothelial cell activation and endothelial–mesenchymal transition have been suggested to occur in the initial phase of the pathology of SSc leading the fibrosis [26,27,28]. In addition to fibroblasts, endothelial cells can differentiate into osteogenic phenotype cells through endothelial–mesenchymal transition induced by TGF-β, TNF-α, and IL-1 [29,30]. Moreover, endothelial–mesenchymal transition is induced by hypoxia and oxidative stress, which is proposed as a contributing factor to the fibrotic process in SSc [31,32]. However, reactive oxygen species (ROS) levels in fibrotic skin and skin without fibrosis from dcSSc patients are significantly elevated compared to skin from healthy participants [33]. Persistent ischemia and reperfusion injury, which ensues as a consequence of vasculopathy in systemic sclerosis (SSc), results in the excessive production of reactive oxygen species (ROS) [34]. It is a well-known fact that oxidative stress plays a crucial role in the development of vascular calcification [35]. A meta-analysis has indicated an increase in levels of several ROS markers and a decrease in antioxidant markers in SSc patients [36]. The study evaluating the skin of SSc patients has brought attention to the fact that the expression level of glucose transporter molecule-1 (GLUT-1) protein, which serves as an indicator of cellular hypoxia, is considerably elevated in the skin of lcSSc patients with calcinosis in contrast to lcSSc patients without calcinosis [37]. Furthermore, hypoxia leads to the accumulation of advanced glycation end products (AGEs) and the activation of receptors of AGEs (RAGEs) triggering oxidative stress, endothelial–mesenchymal transition, vascular damage, and ultimately calcification [38]. In SSc, an elevated expression of AGEs and RAGEs in the skin of lcSSc patients with calcinosis and increased levels of RAGEs from patients with digital ischemic manifestations have been observed [39,40]. The results from clinical studies on SSc have revealed a remarkable association between calcinosis and chronic ischemia manifestations such as digital ulcers, gangrene, and loss of digital pulp [4,41,42]. All these reports of in vitro and clinical studies suggest that hypoxia, excessive ROS production, and endothelial–mesenchymal transition may lead to the development of calcinosis in SSc.

### 2.2. Local Increase in Calcium and Phosphate and Imbalance Between Mineralization Factors

Acro-osteolysis, characterized by the bone resorption of distal phalanges, represents a devastating complication of SSc. Vascular hypoxia and ischemia are considered that the culprit factors contributing to osteolysis [43,44,45,46]. Hypoxia is an important determinant of the formation and activation of osteoclasts, the primary cells responsible for bone resorption leading to the release of calcium and phosphate [47]. The tendency of osteoclastogenesis is heightened in SSc patients with acro-osteolysis [48]. The results from clinical studies have elicited a remarkable relationship between acro-osteolysis and calcinosis in SSc [3,49]. Although studies linking osteoclast activation and calcinosis in SSc are lacking, osteolysis and osteoclast activation induced by hypoxia may lead to a local increase in calcium (Ca^+2^) and phosphate (PO_4_^3−^) levels, thereby promoting extracellular mineralization.

The imbalance between the promoter and inhibitor of mineralization may induce the development of calcinosis in SSc [50]. Osteonectin (called SPARC), an extracellular bone protein linking calcium and type I collagen, has a role in the mineralization of tissues as a promoter and induces the fibrotic process via TGF-B signaling in SSc [51,52]. The serum level of osteonectin was found to be significantly elevated in lcSSc patients compared to healthy controls, while this elevation was not observed in dcSSc patients [53]. An interesting study evaluating the expression of osteonectin also demonstrated an increased expression of osteonectin in endothelial cells and fibroblasts extracted from the skin of lcSSc patients compared to healthy control. Moreover, this expression was more frequently detected in both cell types from lcSSc patients with calcinosis compared to those without calcinosis [54]. Osteopontin, another regulatory bone protein, has a dual effect on mineralization. Furthermore, osteopontin participates in the regulation of the immune system by activating inflammation and tissue remodeling [55,56]. Of interest is the fact that serum osteopontin levels were found to be increased in SSc patients. However, no meaningful difference in osteopontin levels regarding the presence of calcinosis in SSc patients was observed [57]. Osteoprotegerin acts on bone mineralization by reducing osteoclast activation through the blockage of the receptor activation of nuclear factor-B ligand (RANKL) to RANK [58]. Studies assessing the level of osteoprotegerin have shown marked higher levels in lcSSc patients and SSc patients compared to healthy controls [59,60]. However, another study did not find any significant increase in the levels of osteoprotegerin in SSc patients in comparison with healthy participants. This study reported a notably high level of osteoprotegerin in SSc patients with calcinosis compared to patients without calcinosis [61].

Fetuin-A, a regulatory circulating glycoprotein, exerts an inhibitory effect on calcification by binding and stabilizing mineral salts and reducing osteoblastic cell differentiation [50]. A study with a small number of SSc patients demonstrated markedly lower serum fetuin-A levels in SSc patients with calcinosis and lcSSc patients [62]. Extracellular inorganic pyrophosphate (PPi) is involved in the crucial control step of calcification by binding the crystalloid, thus inhibiting the growth of hydroxyapatite formation. The homeostasis between PPi and inorganic phosphate, which is the main contributor of hydroxyapatite, is important for the prevention of ectopic calcinosis [63,64]. Serum PPi levels were decreased in SSc patients compared to healthy controls, while no significant association was found between serum PPi levels and the presence of calcinosis [65].

### 2.3. Extracellular Matrix as a Template and Suitable Microenvironment for Calcinosis Development

The extracellular matrix (ECM) plays a pivotal role in the process of biomineralization, and collagen fibers in ECM serve as a template for the formation of calcium phosphate crystals, which then aggregate into hydroxyapatite, particularly in bone tissue [66]. Moreover, considering its scaffolding role, the ECM has a remarkable impact on hydroxyapatite deposition and growth through regulating mineralization [67].

The ECM is a fundamental player in SSc pathogenesis as the excess in ECM production is the hallmark of the disease, mainly due to the deposition of collagen type I fibers produced by activated fibroblasts called myofibroblasts [68]. The histopathologic examination of calcinosis deposits from SSc patients has demonstrated disorganized collagen fibers and elastin, an increase in the number of fibrocytes, and fibrotic changes, around the calcinosis [9]. In SSc, the fibrotic process results in the stiffness of affected tissue, clinically manifesting as interstitial lung disease, cardiac fibrosis, and skin involvement. The stiffness of the ECM as well as ECM proteins enhance the osteogenic differentiation of MSCs [69,70]. An in vitro study has revealed that MSCs from the skin of dcSSc patients increased osteogenic differentiation capacity under a stiff ECM by stimulating IL-31 [71]. IL-31 is a profibrotic cytokine originating from T helper 2 cells, playing a major role in collagen synthesis and tissue mineralization. Elevated levels of IL-31 have been observed in the sera and skin of SSc patients [72,73]. Moreover, mechanical stress induces the mRNA expression of bone morphogenetic protein-2 (BMP-2), which is the principal activator of Runx2, in endothelial cells and fibroblasts [74].

**Figure 1:**
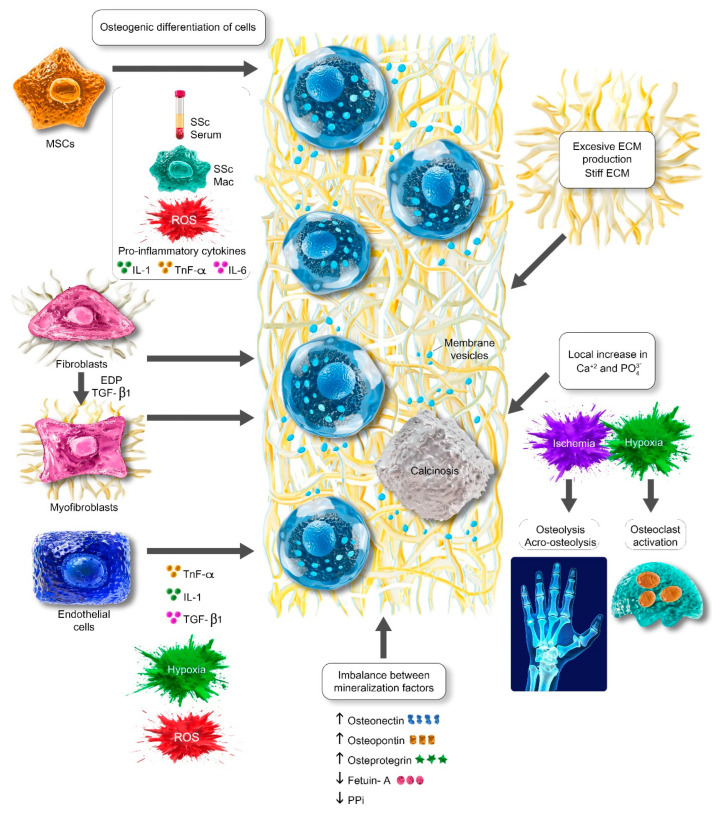
The illustration of potential contributing factors for dystrophic calcinosis in SSc. MSCs derived from SSc patients have osteogenic differentiation capacity when influenced by SSc sera and macrophages, reactive oxidative stress, and pro-inflammatory cytokines. Fibroblasts and myofibroblasts derived from fibroblasts induced by elastin-derived peptides and TGF-β1 can undergo osteogenic differentiation. Endothelial cells can differentiate into osteogenic phenotype when induced by TGF-β, TNF-α, IL-1, ROS, and hypoxia. Hypoxia leads to the activation of osteoclast, which is responsible for bone resorption, thus resulting in a local increase in Ca^+2^ and PO_4_^3−^. Ischemia and hypoxia play an important role in the osteolysis process, and acro-osteolysis is related to the presence of calcinosis in SSc. The levels of mineralization promoters such as osteonectin, osteopontin, and osteoprotegerin increase in SSc patients, whereas the levels of fetuin-A and PPi, considered mineralization inhibitors, are found to decrease. ECM serves as a template for crystal growth and mineralization. Evidence from clinical studies has suggested that excessive ECM or stiff ECM, which is the hallmark of SSc, may contribute to developing calcinosis. Ca^+2^: calcium; ECM: extracellular matrix; IL: interleukin; Mac: macrophages; PPi: inorganic pyrophosphate; PO_4_^3−^: phosphate; ROS: reactive oxygen species; SSc: systemic sclerosis; TGF-β: transforming growth factor-beta; TNF-α: tumor necrosis factor-alpha.

The stiffness of the microenvironment and mechanical pressure hypothesis on calcinosis may be reinforced by reports of clinical studies in SSc. The higher modified Rodman skin score indicating the severity of skin involvement is associated with the presence of calcinosis [75]. In SSc, the localization of calcinosis is more commonly reported in the hand, especially fingertips (thumbs or index finger of the dominant hand), as well as the forearm, knee, and hips, which might be more exposed to pressure or microtrauma [76,77]. These clinical lines of evidence suggest that exaggerated ECM production resulting in the stiffness of the tissue may potentially facilitate calcinosis in SSc (Figure 1). However, further research is warranted to investigate and substantiate this hypothesis.

### 2.4. Membrane Vesicles and DRP-1

During biologic mineralization, extracellular membrane vesicles, derived from cells such as osteoblasts or chondroblasts, form the initial step for mineralization. These vesicles, called matrix vesicles, contain extracellular calcium ions and inorganic phosphate, which are required to form hydroxyapatite crystals. Moreover, matrix vesicles have been observed in ectopic calcification [78]. Notably, apoptotic bodies, which are derived from dying cells, are also another member of membrane vesicles. Both membrane vesicles have been implicated in vascular calcification [79]. Unfortunately, no clear connection between membrane vesicles and dystrophic calcinosis in connective tissue disorders has been identified yet.

Mitochondria, a unique organelle, is responsible for cellular homeostasis and survival. Among their functions, mitochondria are also involved in calcium hemostasis and calcification process in the cell [80]. During bone formation, intracellular calcium phosphate crystals derived from mitochondrial granules participate in apatite formation in addition to extracellular membrane vesicles [81]. Moreover, mitochondria also have an impact on the calcification process by inducing tissue injury through excessive oxidative stress and apoptosis [82].

Mitochondria’s fission and fusion processes are crucial for cell integrity and proliferation [83]. An excessive or abnormal fission process in mitochondria leads to an increase in the production of reactive oxygen species, the hyperproliferation of cells, and mitophagy, thus resulting in cell death [84]. The mitochondrial fission process is mainly regulated by dynamin-related protein-1 (DRP-1), which serves as the cornerstone of the division. DRP-1, compromising three domains with GTPase activity, binds on the outer membrane of mitochondria [85]. DRP-1 is actively involved in the vascular calcification process, and the suppression of DRP-1 leads to a reduction in the differentiation of cells into osteogenic phenotype, collagen synthesis, and tissue calcification [86,87]. Furthermore, DRP-1 is associated with ischemia–reperfusion injury and the hyperproliferation of smooth muscle cells induced by hypoxia or lipopolysaccharides [85,88,89]. Moreover, several studies have demonstrated an elevated DRP-1 expression in pulmonary arterial hypertension [90]. The impact of mitochondrial fission and DRP-1 on the calcification process, along with their role in ischemia and inflammation, prevalent in SSc, suggests their potential involvement in the development of calcinosis in SSc. Therefore, investigating mitochondrial dynamics and their effects on calcinosis in SSc may provide valuable insights into understanding the exact pathogenesis of dystrophic calcification in SSc. The major pathogenic drivers of calcinosis in systemic sclerosis are summarized in Table 1.

## 3. Clinical Aspects of Calcinosis in Systemic Sclerosis

### 3.1. The Localization and Clinical Features of Calcinosis in Systemic Sclerosis

Dystrophic calcinosis in SSc is frequently observed in the upper extremities, especially distal phalanges of hands and elbows and the dorsal part of the knees. The common point of these affected areas is more exposure to pressure and repetitive trauma, which is implicated in contributing factors for the development of calcinosis, mentioned above [3,91]. Additionally, although less commonly, calcinosis may also manifest in the spinal part of the body; hip; and facial regions such as an orbital wall, maxillary sinus, and temporomandibular joints [3,92,93,94,95].

The exact prevalence of calcinosis in SSc remains undetermined, mainly due to its mostly subclinical presentation and asymptomatic progression. The frequency of calcinosis is reported to be higher when detected through imaging modalities compared to clinical examination, which can explain the frequency of calcinosis with a wide range in SSc [96]. In SSc, calcinosis typically presents as small subcutaneous nodules. However, large, calcified masses, known as pseudotumoral calcinosis, can develop in SSc patients. Pseudotumoral calcinosis has been reported in approximately 3% of SSc patients. The size of pseudotumoral calcinosis varies between 2 and 12 cm, with the most frequently affected area being the upper limbs, particularly the hand and wrist [97].

According to Baulman et al., soft tissue calcification can be divided into five distinct subgroups, namely idiopathic; tumoral; metastatic; calciphylaxis; and dystrophic calcification, also referred to as dystrophic calcinosis, which is usually defined in connective tissue disorders, including dermatomyositis, systemic lupus erythematosus, and SSc [98]. Although a higher prevalence of calcinosis has been observed in SSc, only one study including SSc patients with 316 calcinoses has described and classified calcinosis according to palpation/visual and X-ray examination. In this study, calcinosis was divided into four subsets based on morphology (consistency and form) of calcification: stone (with hard consistency), mousse (creamy consistency), plate (palpable as a large, smooth, and uniform agglomerate) and net (physically felt as a thin and diffuse network). Fifty-three percent of calcinoses were not visible but were palpable, and most of them had stone morphology. According to X-ray assessment, the most observed subtype of calcinosis was stone/mousse (%91) in SSc patients [76].

Numerous studies in the literature have examined the relationship between calcinosis and clinical features of SSc. Clinical manifestations of vasculopathy in SSc, such as digital ischemia findings and late capillaroscopic patterns, are considered significant risk factors for calcinosis [4,41,99,100,101]. These clinical lines of evidence have suggested that vasculopathy-related ischemia may be a strong contributing factor in the development of calcinosis in SSc. Additionally, calcinosis has been more frequently detected in SSc patients with older age or longer disease duration [41,100,102]. Although there have been various conflicting results, calcinosis has been found to be associated with the positivity of anti-centromere, anti-RNA polymerase III, and anti-PM/Scl antibodies, which are specific antibodies providing insights into the disease progression in SSc [41,100,103,104]. Another controversial research area related to SSc is the link between the bone mineralization regulatory system and calcinosis in SSc. However, several clinical reports have shown that osteoporosis is independently related to calcinosis [100,102,105].

### 3.2. Complications of Calcinosis in Systemic Sclerosis

From SSc patients’ perspective, calcinosis may have a multifaceted influence on their lives. Functional disability, which directly impacts life, is a significant concern for SSc patients [106]. A study involving 121 patients with SSc has demonstrated that the presence of calcinosis is related to functional disability [107]. Another report evaluating SSc patients from Bangladesh has shown that those with calcinosis have increased functional impairment and a decreased quality of life [108]. Furthermore, a result of a multi-center cohort study has indicated that calcinosis markedly affects hand function and leads to greater disability. Also, the presence of calcinosis is reported to be an independent determinant of functional impairment [4].

Another main complication of SSc is intractable pain, which may usually be disregarded by physicians. Approximately one-third of SSc patients experience moderate or severe pain [6]. Moreover, pain is one of the main risk factors for disability and reduced quality of life in SSc patients [109]. Several factors are responsible for pain in SSc, with calcinosis being one of the primary causes. SSc patients with calcinosis have a higher degree of pain, and calcinosis is a major risk factor for pain [4]. Moreover, pseudotumoral calcinosis results in pain and functional disabilities in most SSc patients [97]. When calcinosis is present in the paraspinal region of the body, calcinosis might lead to neurologic manifestations as a result of compression [110,111]. Calcinosis also causes peripheral neuropathy and is considered to be the most prevalent cause of compression-related neuropathy [112].

The development of calcinosis-related skin ulcers is a common complication, occurring in approximately half of the SSc patients with calcinosis, and these ulcers have a prolonged healing time [5,76,113,114]. Calcinosis may lead to infection, which manifests as pain and tenderness and sometimes presents systemic signs such as fever and chills [115,116].

### 3.3. Treatment Modalities of Calcinosis in Systemic Sclerosis

The treatment of calcinosis in SSc remains another area of debate due to the limited evidence, which is primarily based on expert opinions and clinical reports derived from observational non-randomized controlled studies or case series. The general aim of the treatment strategy for calcinosis is usually to prevent complications of calcinosis such as infection, pain, physical disability, or compression-related symptoms [91,117].

Numerous medications, including calcium channel blockers, colchicine, minocycline, and bisphosphonates, have been utilized in calcinosis treatment, with some results indicating improvement or a reduction in calcinosis associated with their use [3,91,96]. Additionally, the use of immunomodulatory drugs such as leflunomide, rituximab, tofacitinib, or tumor necrosis factor-alpha inhibitor has shown potential in improving calcinosis in SSc, as supported by evidence from case reports and case series [118,119,120,121].

Topical or intralesional therapies represent another treatment option for calcinosis in SSc. The use of topical Holoil, containing neem oil and Hypericum perforatum, has beneficial effects on calcinosis-related skin ulcers [122]. Topical or intralesional sodium thiosulfate has been effective for an improvement in calcinosis [123,124]. Surgical treatment is frequently preferred in refractory cases or patients with calcinosis who also present infection, compression signs, or physical disabilities [125,126,127].

## 4. Conclusions

In summary, calcinosis represents a serious problem in SSc due to its potential complications, including functional impairment, a decrease in health-related quality of life, pain, ulceration, compression-related symptoms, and infection. Therefore, the definitive detection of calcinosis and its related complications in SS becomes crucial for early intervention. Although the exact pathogenesis remains elusive, numerous processes observed in SSc, including the osteogenic differentiation of cells, local disturbance in calcium and phosphate, an increase in the promotion of mineralization, a reduction in the inhibition of mineralization, and excessive extracellular matrix production might be implicated. Furthermore, ischemia/hypoxia, excessive production of reactive oxygen species and inflammatory cytokines, and mechanical stress may be considered promoters of calcinosis development in SSc. Moreover, recent evidence has highlighted a potential link between mitochondrial dynamics, especially mitochondrial fission and DRP-1, and the calcinosis process in SSc. Understanding these complex mechanisms can ensure the development of new and improved management and treatment modalities for SSc patients with calcinosis.

## Figures and Tables

**Table 1:** Major factors implicated in calcinosis in systemic sclerosis and their biological effect.

Factor	Biological Effect	Clinical Significance in Calcinosis in Systemic Sclerosis
Osteonectin (SPARC)	Promotes mineralization and induces fibrotic processes via TGF-B [51,52]	Increased expression in fibroblasts and endothelial cells of patients with calcinosis
Osteopontin	Regulates mineralization and immune activation [55,56]. No significant link to calcinosis in SSc [57]	Increased serum levels in SSc but no association with calcinosis specifically
Osteoprotegerin	Reduces osteoclast activation through the blockage of receptor activation of nuclear factor-B ligand (RANKL) to RANK [58]	Significant in SSc patients with calcinosis, not elevated in all SSc patients
Fetuin-A	Inhibits calcification by stabilizing mineral salts [50,62]	Lower serum levels associated with calcinosis and lcSSc patients
Extracellular Pyrophosphate (PPi)	Regulates calcification through hydroxyapatite inhibition [63,64]	Imbalance contributes to ectopic calcification, with lower levels seen in SSc
Extracellular matrix (ECM)	Serves as a template for hydroxyapatite, fibrosis increases ECM stiffness, which facilitates calcinosis [66,67]	Excess ECM production promotes calcinosis, especially in high-pressure areas
IL-31	Profibrotic cytokine. Enhances osteogenic differentiation under stiff ECM conditions [71]	Elevated in SSc, associated with increased osteogenic differentiation and fibrosis
Membrane Vesicles	Observed in ectopic calcification [78] Matrix vesicles from dying cells initiate mineralization	No direct evidence connecting vesicles to calcinosis in SSc
Mitochondria and DRP-1	Regulates mitochondrial fission, linked to vascular calcification, promotes apoptosis and oxidative stress [80,81]	Implicated in the development of calcinosis in SSc
